# Summary of the National Advisory Committee on Immunization (NACI) Seasonal Influenza Vaccine Statement for 2023–2024

**DOI:** 10.14745/ccdr.v49i10a01

**Published:** 2023-10-01

**Authors:** Angela Sinilaite, Winnie Siu, Jesse Papenburg

**Affiliations:** 1 Centre for Immunization Programs, Public Health Agency of Canada, Ottawa, ON; 2 School of Epidemiology and Public Health, Faculty of Medicine, University of Ottawa, ON; 3 NACI Influenza Working Group Chair; 4 Division of Pediatric Infectious Diseases, Department of Pediatrics, Montréal Children’s Hospital of the McGill University Health Centre, Montréal, QC; 5 Division of Microbiology, Department of Clinical Laboratory Medicine, Optilab Montréal - McGill University Health Centre, Montréal, QC; 6 Department of Epidemiology, Biostatistics, and Occupational Health, School of Population and Global Health, McGill University, Montréal, QC

**Keywords:** National Advisory Committee on Immunization, NACI, influenza, influenza vaccine, guidance

## Abstract

**Background:**

The National Advisory Committee on Immunization (NACI) reviews the evolving evidence on influenza immunization and provides annual recommendations regarding the use of seasonal influenza vaccines. The NACI *Statement on Seasonal Influenza Vaccine for 2023–2024* updates the 2022–2023 NACI recommendations.

**Objective:**

To summarize the 2023–2024 NACI seasonal influenza vaccine recommendations and to highlight new and updated information.

**Methods:**

In the preparation of the *Statement on Seasonal Influenza Vaccine for 2023–2024*, the NACI Influenza Working Group applied the NACI evidence-based process to critically appraise the available evidence and to propose recommendations. The recommendations were then considered and approved by NACI in light of the available evidence.

**Results:**

Key changes for the 2023–2024 season include: 1) incorporation of updated information/guidance on influenza vaccination in the context of the coronavirus disease 2019 (COVID-19); 2) new recommendations for Flucelvax^®^ Quad and Influvac^®^ Tetra, the two quadrivalent inactivated influenza vaccines with expanded paediatric age indications; and 3) an update to the format of the Statement.

**Conclusion:**

Overall, NACI continues to recommend that an age-appropriate influenza vaccine should be offered annually to all individuals aged six months and older who do not have a contraindication to the vaccine, with particular focus on the groups for whom influenza vaccination is particularly recommended.

## Introduction

In Canada, seasonal influenza epidemics generally occur in the late fall and winter months and can lead to significant morbidity and mortality ((1)). The burden of influenza varies from year to year and some groups, including young children (younger than six years of age), older adults (65 years of age and older), people with chronic health conditions, pregnant individuals and Indigenous peoples are at higher risk of experiencing severe illness, complications or worsening of chronic health conditions. Influenza vaccination is a critical tool to mitigate ongoing health system stress through protection against influenza-related disease.

The National Advisory Committee on Immunization (NACI) provides the Public Health Agency of Canada (PHAC) with annual recommendations regarding the use of authorized seasonal influenza vaccines, which reflect identified changes in influenza epidemiology, immunization practises and influenza vaccine products available for use in Canada. The annual update of the NACI statement on seasonal influenza vaccine is led by the NACI Influenza Working Group (IWG) and involves a thorough review and evaluation of the literature as well as discussion and debate at the scientific and clinical practice levels. On May 31, 2023, PHAC released new guidance from NACI on the use of seasonal influenza vaccines for the 2023–2024 season, which is based on current evidence and expert opinion. This article provides a concise summary of NACI’s recommendations and supporting information for the 2023–2024 influenza season, including conclusions from evidence reviews on two quadrivalent inactivated influenza vaccines with expanded age indications in children six months of age and older. Updated NACI guidance on concurrent administration of influenza vaccines with the coronavirus disease 2019 (COVID-19) vaccines is also highlighted. Complete details are available in the new NACI Advisory Committee *Statement on Seasonal Influenza Vaccine for 2023–2024* (the Statement) on the PHAC website ((2)).

## Methods

When preparing the *Statement on Seasonal Influenza Vaccine for 2023–2024*, the NACI IWG identified the need for evidence reviews for new topics, reviewed and analyzed the available evidence, and proposed updated recommendations according to the NACI evidence-based process for developing recommendations ((3)). Further details regarding the strength of NACI recommendations are available in **Table A1** in the **Appendix**. NACI’s peer-reviewed framework and evidence-informed tools (including the Ethics Integrated Filters, Equity Matrix, Feasibility Matrix, and Acceptability Matrix) were applied to help ensure that issues related to ethics, equity, feasibility and acceptability are systematically assessed and integrated into NACI guidance ((4)).

## Results

### New or updated information for 2023–2024

The 2023–2024 Statement includes updated information and guidance on influenza in the context of COVID-19, including an overview of changes in influenza epidemiology over the course of the COVID-19 pandemic and updated content on concurrent administration of influenza vaccines with COVID-19 vaccines. NACI guidance states that administration of COVID-19 vaccines may occur at the same time as, or at any time before or after, influenza immunization (including all parenteral and intranasal seasonal influenza vaccines) for individuals six months of age and older. Updated NACI guidance and additional information on concurrent administration of COVID-19 vaccines with influenza vaccines and across all eligible age groups is available in the COVID-19 vaccines: Canadian Immunization Guide chapter ((5)).

For the 2023–2024 influenza season, NACI reviewed the available evidence and developed updated recommendations for:

1) Flucelvax^®^ Quad, a mammalian cell culture-based influenza vaccine (IIV4-cc)

2) Influvac^®^ Tetra, an egg-based, standard dose influenza vaccine (IIV4-SD)

NACI provided the following new recommendations based on a review and analysis of Health Canada assessments of supporting clinical trial evidence submitted by the manufacturers:

1) **NACI recommends that Flucelvax Quad may be considered among the quadrivalent influenza vaccines offered to adults and children six months of age and older (Discretionary NACI Recommendation)**

2) **NACI recommends that Influvac Tetra may be considered among the standard dose inactivated quadrivalent influenza vaccines offered to individuals three years of age and older (Discretionary NACI Recommendation)**

At this time, NACI concludes that there is insufficient evidence for recommending vaccination with Influvac Tetra in children younger than three years of age (Discretionary NACI Recommendation).

NACI will continue to monitor the evidence as it emerges, and update recommendations as needed. To improve readability and access to information, the format and structure of the Statement has been updated from previous seasons’ statements. Notably, clinical information on seasonal influenza vaccine administration for vaccine providers is now contained in the new Influenza vaccines chapter of the Canadian Immunization Guide ((6)).

Summary of National Advisory Committee on Immunization recommendations for the use of influenza vaccines for the 2023–2024 influenza season

NACI continues to recommend influenza vaccination to anyone six months and older who does not have a contraindication to the vaccine. Vaccination should be offered as a priority to people at high risk of influenza-related complications or hospitalization, people capable of transmitting influenza to those at high risk of complications, and others as indicated in **List 1**.

### List 1: Groups for whom influenza vaccination is particularly recommended

People at high risk of influenza-related complications or hospitalization

• All children 6–59 months of age

• Adults and children with the following chronic health conditions^a^:

o Cardiac or pulmonary disorders (includes bronchopulmonary dysplasia, cystic fibrosis and asthma)

o Diabetes mellitus and other metabolic diseases

o Cancer, immune compromising conditions (due to underlying disease, therapy, or both, such as solid organ transplant or hematopoietic stem cell transplant recipients)

o Renal disease

o Anemia or hemoglobinopathy

o Neurologic or neurodevelopment conditions (includes neuromuscular, neurovascular, neurodegenerative, neurodevelopmental conditions and seizure disorders [and, for children, includes febrile seizures and isolated developmental delay], but excludes migraines and psychiatric conditions without neurological conditions)^b^

o Morbid obesity (body mass index of 40 kg/m^2^ and over)

o Children six months to 18 years of age undergoing treatment for long periods with acetylsalicylic acid, because of the potential increase of Reye’s syndrome associated with influenza

• All individuals who are pregnant

• People of any age who are residents of nursing homes and other chronic care facilities

• Adults 65 years of age and older

• Indigenous peoples

People capable of transmitting influenza to those at high risk

• Healthcare and other care providers in facilities and community settings who, through their activities, are capable of transmitting influenza to those at high risk

• Household contacts, both adults and children, of individuals at high risk, whether or not the individual at high risk has been vaccinated:

o Household contacts of individuals at high risk

o Household contacts of infants less than six months of age, as these infants are at high risk but cannot receive influenza vaccine

o Members of a household expecting a newborn during the influenza season

• Those providing regular childcare to children 0–59 months of age, whether in or out of the home

• Those who provide services within closed or relatively closed settings to people at high risk (e.g. crew on a cruise ship)

Others

• People who provide essential community services

• People who are in direct contact with poultry infected with avian influenza during culling operations

Recommended influenza vaccine options by age group, and recommended dose and route of administration of influenza vaccine types by age, are summarized in **Table 1** and **Table 2** respectively.

## Conclusion

NACI continues to recommend annual influenza vaccination for all individuals aged six months and older (noting product-specific age indications and contraindications). Influenza vaccination is particularly important for people at high risk of influenza-related complications or hospitalization; people capable of transmitting influenza to those at high risk; people who provide essential community services; and people in direct contact during culling operations with poultry infected with avian influenza. For the 2023–2024 influenza season, NACI advises that: 1) Flucelvax^®^ Quad may be considered among the quadrivalent influenza vaccines offered to adults and children six months of age and older and 2) Influvac^®^ Tetra may be considered among the standard dose inactivated quadrivalent influenza vaccines offered to individuals three years of age and older.

## Appendix

**Table A1:** Strength of the National Advisory Committee on Immunization recommendations

Strength of NACI recommendation (based on factors not isolated to strength of evidence, e.g. public health need)	Strong	Discretionary
Wording	“**should/should not** be offered”	“**may be** considered”
Rationale	Known/anticipated advantages outweigh known/anticipated disadvantages (“should”), OR known/anticipated disadvantages outweigh known/anticipated advantages ("should not")	Known/anticipated advantages closely balanced with known/anticipated disadvantages, OR uncertainty in the evidence of advantages and disadvantages exists
Implication	A strong recommendation applies to most populations/individuals and should be followed unless a clear and compelling rationale for an alternative approach is present	A discretionary recommendation may be considered for some populations/individuals in some circumstances Alternative approaches may be reasonable

Abbreviation: NACI, National Advisory Committee on Immunization

## Figures and Tables

**Table 1:** Recommendations on choice of influenza vaccine type for individual and public health program-level decision making by age group

Recipient by age group	Vaccine types authorized^a,b^ for use	Recommendations on choice of influenza vaccine	
6–23 months	IIV3-Adj IIV4-SD IIV4-cc	• A quadrivalent influenza vaccine licensed for this age group should be used in infants and young children without contraindications, given the burden of influenza B disease in this age group and the potential for lineage mismatch between the predominant circulating strain of influenza B and the strain in a trivalent vaccine o Currently, there is insufficient evidence for recommending vaccination with Influvac Tetra (IIV4-SD) in children younger than 3 years of age • If a quadrivalent vaccine is not available, a trivalent vaccine licensed for this age group should be used	
2–17 years^c^	IIV4-SD IIV4-cc LAIV4	• An age-appropriate quadrivalent influenza vaccine (IIV4-SD, IIV4-cc or LAIV4) should be used in children without contraindications or precautions (see text below applicable to LAIV), including those with chronic health conditions, given the burden of influenza B disease in this age group and the potential for lineage mismatch between the predominant circulating strain of influenza B and the strain in a trivalent vaccine o Currently, there is insufficient evidence for recommending vaccination with Influvac Tetra (IIV4-SD) in children younger than 3 years of age • LAIV4 may be given to children with: o Stable, non-severe asthma o Cystic fibrosis who are not being treated with immunosuppressive drugs (e.g. prolonged systemic corticosteroids) o Stable HIV infection, if the child is currently being treated with ART (i.e. HAART) and has adequate immune function • LAIV should not be used in children or adolescents for whom it is contraindicated or for whom there are warnings and precautions such as those with: o Severe asthma (defined as currently on oral or high dose inhaled glucocorticosteroids or active wheezing) o Medically attended wheezing in the seven days prior to vaccination o Current receipt of aspirin or aspirin-containing therapy o Immune compromising conditions, with the exception of stable HIV infection (i.e. if the child is treated with HAART for at least 4 months and has adequate immune function) o Pregnancy - In pregnancy, IIV4-SD or IIV4-cc should be used instead	
18–59 years	IIV4-SD IIV4-cc RIV4 LAIV4	• Any of the available influenza vaccines authorized for this age group should be used in adults 18–59 years of age without contraindications or precautions, noting the following consideration and exceptions: o There is some evidence that IIV may provide better efficacy than LAIV in healthy adults • LAIV is not recommended for: o Pregnant individuals - In pregnancy, IIV4-SD, IIV4-cc or RIV4 should be used instead o Adults with any of the chronic health conditions identified in List 1, including immune compromising condition o Healthcare workers	
60–64 years	IIV4-SD IIV4-cc RIV4	• Any of the available influenza vaccines authorized for this age group should be used in adults 60–64 years of age without contraindications	
65 years and older^d^	IIV3-Adj IIV4-SD IIV4-HD IIV4-cc RIV4	**Individual-level decision-making**	**Public health program-level decision-making**
		• IIV-HD should be used over IIV-SD, given the burden of influenza A(H3N2) disease and the good evidence of IIV3-HD providing better protection compared to IIV3-SD in adults 65 years of age and older o Other than a recommendation for using IIV-HD over IIV-SD formulations, NACI has not made comparative individual-level recommendations on the use of the other available vaccines in this age group. In the absence of a specific product, any of the available age-appropriate influenza vaccines should be used	• Any of the available influenza vaccines authorized in this age group should be used o There is insufficient evidence on the incremental value of different influenza vaccines (i.e. cost-effectiveness assessments have not been performed by NACI) to make comparative public health program-level recommendations on the use of the available vaccines

Abbreviations: ART, antiretroviral therapy; HAART, highly active antiretroviral therapy; IIV, inactivated influenza vaccine; IIV3-Adj, adjuvanted trivalent inactivated influenza vaccine; IIV3-HD, high-dose trivalent inactivated influenza vaccine; IIV4-cc, quadrivalent mammalian cell-culture based inactivated influenza vaccine; IIV4-HD, high-dose quadrivalent inactivated influenza vaccine; IIV4-SD, standard-dose quadrivalent inactivated influenza vaccine; LAIV, live attenuated influenza vaccine; LAIV4, quadrivalent live attenuated influenza vaccine; NACI, National Advisory Committee on Immunization; RIV4, quadrivalent recombinant influenza vaccine

^a^ IIV3-SD formulation will not be authorized or available for use in Canada during the 2023–2024 influenza season

^b^ IIV3-HD formulations will not be authorized or available for use in Canada during the 2023–2024 influenza season

^c^ Refer to Table 3 of the NACI *Seasonal Influenza Vaccine Statement for 2023–2024* for a summary of vaccine characteristics of LAIV compared with IIV in children 2–17 years of age

^d^ Refer to Table 4 of the NACI *Seasonal Influenza Vaccine Statement for 2023–2024* for a comparison of the vaccine characteristics of influenza vaccine types available for use in adults 65 years of age and older

Source: Table reproduced from NACI *Seasonal Influenza Vaccine Statement for 2023–2024* ((2))

**Table 2:** Recommended dose and route of administration, by age, for influenza vaccine types authorized for the 2023–2024 influenza season

Age group	Influenza vaccine type (route of administration)						Number of doses required
	IIV4-SD^a^ (IM)	IIV4-cc^b^ (IM)	IIV3-Adj^c^ (IM)	IIV4-HD^d^ (IM)	RIV4^e^ (IM)	LAIV4^f^ (intranasal)	
6–23 months	0.5 mL^g^	0.5 mL	0.25 mL	-	-	-	1 or 2^h^
2–8 years	0.5 mL	0.5 mL	-	-	-	0.2 mL (0.1 mL per nostril)	1 or 2^h^
9–17 years	0.5 mL	0.5 mL	-	-	-	0.2 mL (0.1 mL per nostril)	1
18–59 years	0.5 mL	0.5 mL	-	-	0.5 mL	0.2 mL (0.1 mL per nostril)	1
60–64 years	0.5 mL	0.5 mL	-	-	0.5 mL	-	1
65 years and older	0.5 mL	0.5 mL	0.5 mL	0.7 mL	0.5 mL	-	1

Abbreviations: IIV3-Adj, adjuvanted trivalent inactivated influenza vaccine; IIV4-cc, quadrivalent mammalian cell culture based inactivated influenza vaccine; IIV4-HD, high-dose quadrivalent inactivated influenza vaccine; IIV4-SD, standard-dose quadrivalent inactivated influenza vaccine; IM, intramuscular; LAIV4, quadrivalent live attenuated influenza vaccine; RIV4, quadrivalent recombinant influenza vaccine

^a^ Afluria^®^ Tetra (5 years and older), Flulaval^®^ Tetra (6 months and older), Fluzone^®^ Quadrivalent (6 months and older), Influvac^®^ Tetra (3 years and older)

^b^ Flucelvax^®^ Quad (6 months and older)

^c^ Fluad Pediatric^®^ (6–23 months) or Fluad^®^ (65 years and older)

^d^ Fluzone^®^ High-Dose Quadrivalent (65 years and older)

^e^ Supemtek™ (18 years and older)

^f^ FluMist^®^ Quadrivalent (2–59 years)

^g^ Evidence suggests moderate improvement in antibody response in infants, without an increase in reactogenicity, with the use of full-vaccine doses (0.5 mL) for unadjuvanted inactivated influenza vaccines ((10,11)). This moderate improvement in antibody response without an increase in reactogenicity is the basis for the full dose recommendation for unadjuvanted inactivated vaccine for all ages. For more information, refer to *Statement on Seasonal Influenza Vaccine for 2011–2012* ((12))

^h^ Children six months to less than nine years of age receiving seasonal influenza vaccine for the first time in their life should be given two doses of influenza vaccine, with a minimum interval of four weeks between doses. Children six months to less than nine years of age who have been properly vaccinated with one or more doses of seasonal influenza vaccine in the past should receive one dose of influenza vaccine per season thereafter

Source: Table reproduced from NACI *Seasonal Influenza Vaccine Statement for 2023–2024* ((2))
